# Chloride corrosion behavior on heating pipeline made by AISI 304 and 316 in reclaimed water†

**DOI:** 10.1039/d1ra06695a

**Published:** 2021-12-02

**Authors:** Xi Chen, Hongyan Liu, Xiang Sun, Botao Zan, Meisheng Liang

**Affiliations:** College of Environmental Science and Engineering, Taiyuan University of Technology Taiyuan China liangmeisheng@tyut.edu.cn; University of Washington Seattle 98105 WA USA

## Abstract

In order to transport reclaimed water safely through stainless steel (SS) heat-supply pipeline networks during their idle period, one must understand the degree to which chlorine in reclaimed water is corrosive to SS. In this study, electrochemical methods were used to evaluate the corrosion resistances of two types of SS materials, AISI 304 and AISI 316, in simulated reclaimed water at chloride concentrations of 25 to 400 mg L^−1^, which are similar to those present in practice. The differences in corrosion resistance between the two types of SS material were investigated using electrochemical impedance spectroscopy (EIS) and potentiodynamic polarization tests (Tafel curves). The passivation layers on the two types of SS exhibited obvious similarities under several experimental conditions. However, EIS, polarization resistance, effective capacitance, Tafel curve, and Scanning Electron Microscope (SEM) data showed that AISI 316 has better corrosion resistance than AISI 304. The corrosion behaviours could be described as a series of reactions between Fe, Cr, and H_2_O that generate several precipitated products such as Fe_2_O_3_, Cr_2_O_3_, FeOOH, and CrOOH.

## Abstract 2

It would be economical if heating pipes were used to transport reclaimed water during its idle period. The most important thing is to study the causes and processes of the corrosion on it for practical application.

## Graphical abstract

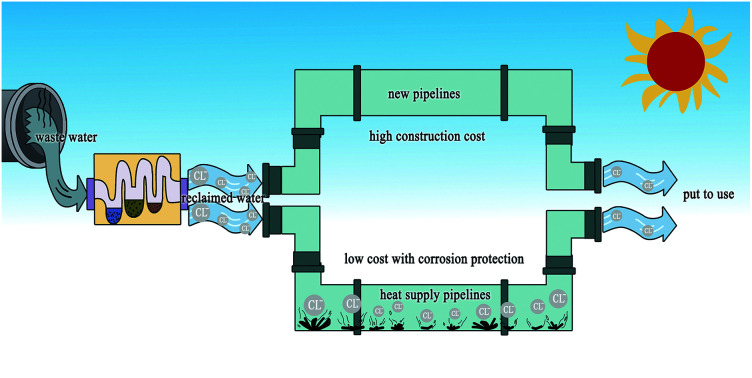

## 1 Introduction

As a new type of renewable resource, reclaimed municipal wastewater is typically used extensively for industrial purposes.^1–3^ However, it is gradually becoming recognised as a secondary urban water source. However, the utilization of reclaimed water suffers from the problem of high water-supply pipeline system construction costs.^4^ There is an idea that widely deployed heat-supply pipeline systems with complete supporting facilities could be used to transport reclaimed water during their idle period (7 months of the year). Thus, it is necessary to ensure that there are no harmful effects associated with this use. Most heat-supply pipelines are made from carbon steel (CS) and stainless steel (SS). CS pipelines typically provide weaker corrosion resistance than SS pipelines during long-running water delivery processes.^5^ This can result in the accumulation of corrosion products on the inner wall and thus affect water quality and safety. Previous studies have revealed that there is no significant precipitation of metal ions in SS pipelines during transportation of reclaimed water.^6,7^ Use of SS pipelines may be a reliable corrosion-protection method capable of guaranteeing good reclaimed water quality.^8^ The most common SS pipeline materials are austenitic SS (AISI 304 and AISI 316), which account for about 70% of all SS use. Although previous works have shown that both AISI 304 and AISI 316 exhibit excellent corrosion resistance in chloride-contaminated media,^9,10^ the complex quality and high salt content of reclaimed water have additional negative effects on corrosion in SS pipelines and partly limit the utilization of SS.^11,12^ The presence of chlorides (Cl^−^) has been demonstrated in previous studies to cause severe damage to passivation layers. It mainly leads to pitting corrosion on the metal media,^13–15^ that reduced transmission efficiency and service life, even caused pipes broke.^16,17^ Furthermore, switching of heat-supply pipeline system water sources between the heating medium and reclaimed water has been confirmed by many studies to be the main cause of the “red water” phenomenon.^18,19^ Therefore, it is especially important to understand the influence of Cl^−^ from reclaimed water on AISI 304 and AISI 316 pipeline corrosion.

The electrochemical technique is a reliable and accurate method of studying the corrosion process. The technique utilises the principle of electrochemical corrosion to simulate the real electrochemical reaction on the metal surface, and then uses a computer to obtain corresponding parameters so that one can analyse the corrosion process and benefit from the advantages of high sensitivity, simple operation, and easy access. Of the several electrochemical techniques available, potentiodynamic polarization tests and electrochemical impedance spectroscopy (EIS) are common, non-disruptive tools for investigating the states of the passivating films formed on metal surfaces.

Over the past few years, numerous studies have considered the characteristics and mechanisms of corrosion of iron and steel components.^20–22^ Well-known studies have focused on corrosion products and scale within water supply system pipelines^23,24^ because of reports that corrosion scale inside pipelines is related to interactions between the pipeline material and water that contains dissolved corrosive substances.^25–27^ In addition, the type of pipeline corrosion must be considered. Corrosion is typically classified as either uniform or localised.^28^ Localised corrosion (including pitting corrosion, crevice corrosion, intergranular corrosion, and stress corrosion cracking) can affect the SS pipeline service life greatly.^29,30^ However, few studies have focused on the corrosion of pipelines in heat supply networks. And there was an idea that what would happen if heating pipes were used to transport reclaimed water during its idle period. Some unpredictable phenomena may occur during transport due to the complex components of reclaimed water. Then the causes and processes of the corrosion on the heating pipes must be studied due to the practical consideration. Cl^−^ is significant factor in pipeline corrosion, but studies have focused mainly on the effects of seawater on steel-reinforced concrete and of desalinated seawater on water pipe systems. These studies are all aimed at high concentrations of Cl^−^, which have limited reference significance to corrosion driven by low-concentration Cl^−^ in a reclaimed water pipeline system.

Based on this, we studied the corrosion behaviours of AISI 304 and AISI 316 in a simulated reclaimed water solution that contained Cl^−^. Chloride ion was provided *via* addition of sodium chloride (NaCl) at various concentrations. Based on the results of electrochemical tests and the influence of Cl^−^ on SS passivation behaviours, we can provide a theoretical basis for the industrial application of SS heat-supply pipeline systems to the transport of Cl^−^ containing reclaimed water during their idle period. Such information has practical application value.

## 2 Experimental section

### 2.1 Material preparation

AISI 304 and AISI 316 samples were supplied by Keli Environmental Protection (Yangzhou, Jiangsu, China). Their chemical compositions are shown in Table 1. The samples were processed into 10 mm × 10 mm × 2 mm (width × length × thickness) squares with 1 cm^2^ working surfaces. A line of copper was welded on one side of each square as an electrochemical performance test electrode. In order to prevent the non-working surface from contacting the corrosive medium, epoxy resin was used to package the back sides of the metal samples. The working surface was ground using a series of silicon carbide (SiC) emery papers from grade 280 to 2000, and then washed with deionised water, acetone, and absolute ethanol to ensure that the surface was clean. Finally, the samples were placed in a desiccator for 24 h after drying in air. Before the electrochemical test, the samples were sterilised using an ultraviolet light for 30 min.

**Table 1:** Chemical composition (wt%) of AISI 304 and AISI 316

Material	C	Mn	Si	P	S	Cr	Ni	Cu	Mo	N	Fe
AISI 304	0.042	1.06	0.47	0.033	0.004	18.02	8.02	—	—	—	Balance
AISI 316	0.017	1.310	0.562	0.029	0.002	16.60	10.01	—	2.01	0.014	Balance

### 2.2 Experiments

The chemical components and quality parameters of the reclaimed water used in the experiments are shown in Table 2. In order to eliminate interference from other factors, laboratory-grade NaCl and deionised water were used to prepare NaCl solutions with various chloride concentrations (25 mg L^−1^, 50 mg L^−1^, 100 mg L^−1^, 200 mg L^−1^, and 400 mg L^−1^) as corrosive media. After preparation, each solution was placed in a constant-temperature water bath to ensure that the reaction occurred at 40 °C. Then, nitrogen (N_2_) was poured into the solution for 30 min to drive oxygen out. The solution was used immediately to avoid contamination.

**Table 2:** Quality parameters of reclaimed water

Items	Value
Cl^−^ (mg L^−1^)	226.7
SO_4_^2−^ (mg L^−1^)	300
HCO_3_^−^ (mg L^−1^)	320
Alkalinity (mg L^−1^)	254
Hardness (mg L^−1^)	670
Ca^2+^ (mg L^−1^)	158.54
Mg^2+^ (mg L^−1^)	59.63
pH	7.24
DO (mg L^−1^)	8.02
NH_3_–N (mg L^−1^)	3.03
Total phosphorus (mg L^−1^)	0.232

**Fig. 1:**
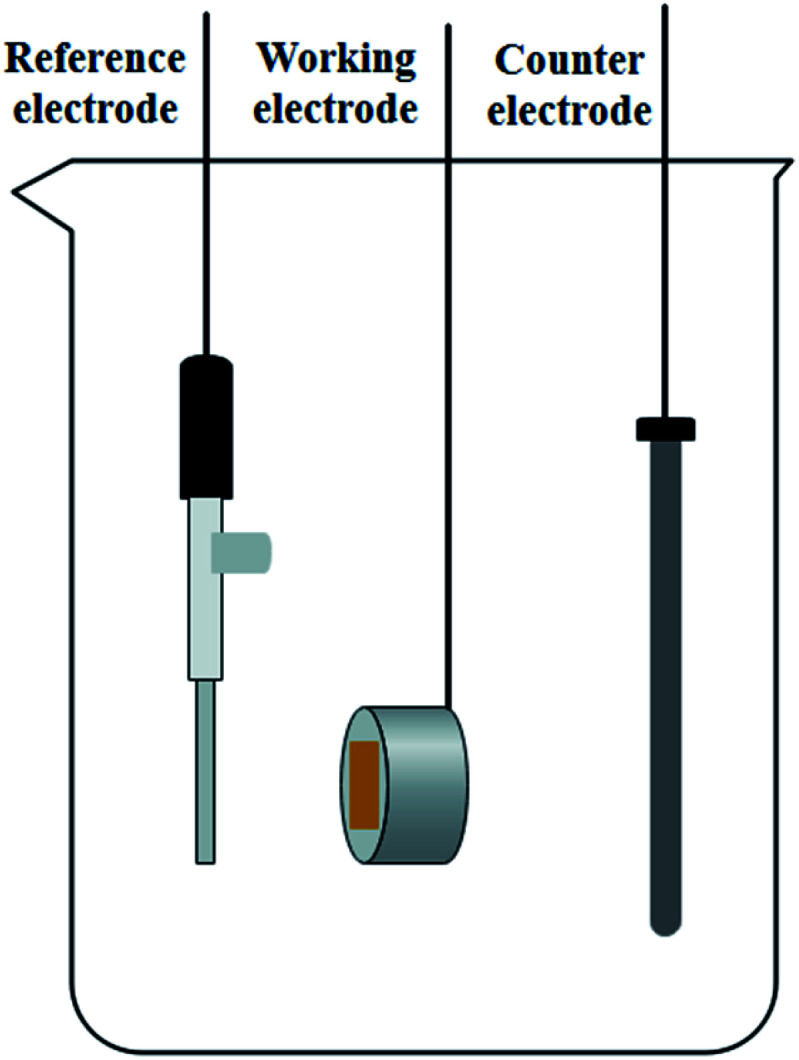
Schematic diagram of the three-electrode test cell.

All electrochemical tests were performed using a conventional three-electrode configuration, with the processed AISI 304 and AISI 316 samples acting as working electrodes, an Ag/AgCl electrode as a reference electrode with the standard potential of 0.198 V (25 °C), and a graphite rod as the counter electrode. The test cell of the three-electrode configuration is shown schematically in Fig. 1. During the experiments, a CHI760E (Shanghai Chenhua Instruments) electrochemical workstation was used to perform the electrochemical tests. The morphologies of the two types of SS were observed using a scanning electron microscope (SEM, LYRA3, TESCAN) produced by TESCAN. Details of the electrochemical tests are shown in S1.†

## 3 Results and discussion

### 3.1 Electrochemical Impedance Spectroscopy (EIS)

**Fig. 2:**
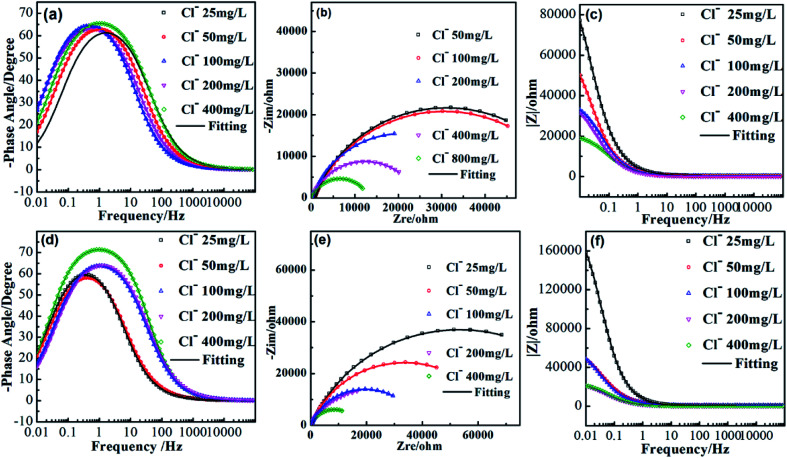
EIS spectra for AISI 304 (a–c) and AISI 316 (d–f) in simulated solutions.

Fig. 2 shows the EIS spectra of AISI 304 and AISI 316 in simulated solutions with various chloride concentrations. According to their Nyquist and Bode plots, AISI 304 and AISI 316 exhibit similar corrosion behaviours and characteristics. In EIS, a potential (or current) with a small-amplitude sinusoidal wave presents firstly as an interference signal. Then, the impedance could be calculated according to the wave frequency (*f*) and phase angle (*ϕ*). Finally, the impedance (*Z*) of the electrode is obtained by measuring the ratio of the interference signal to the response signal.

In Fig. 2a and d, it can be seen clearly that the maximum phase angle is smaller than 90°. The phase angles (*ϕ*) are maintained at zero degrees under high-frequency conditions due to resistive behaviour, rather than a relaxation process. The phenomenon appears to present a capacitive-like behaviours, where the phase angle maximum is in the position of the medial frequencies. At the lowest values, the phase angle gradually approaches zero. This suggests that |*Z*| shown in Fig. 2c and f could be recognized as the characteristic resistance. The raw experimental results indicate that this system seems to be defined by a single resistance process.

A type of EIS spectrum, the Nyquist plot is drawn with the real part of *Z* (*Z*_re_) on the horizontal axis and the imaginary part of *Z* (*Z*_im_) on the vertical axis. The interfacial layer between the working electrode and the electrolyte is regarded as a type of circuit element. The imaginary part of the impedance is shown as a circular arc in the first quadrant of the impedance spectrum, and is known as the capacitive arc. In Fig. 2b and e, for the two samples, the radians of the curves present the similar trends in solutions with different chloride concentrations. The radius of the capacitive arc can reflect the status of charge transfer and can indirectly imply the corrosion resistance of the working electrode. Comparison of the Nyquist plots in Fig. 2b and e indicates that both SS materials produce imperfect semicircles without straight lines in the low-frequency range. The phenomenon could be explained as the incomplete diffusion of chloride.^31^ When the chloride concentration is increased, there is a remarkable increase in the diameter of the capacitive arc. This can be understood as a change in corrosion behaviour in the presence of chloride.^32^ The charge-transfer resistance also increases accordingly, resulting in the emergence of more stable passivation films with stronger corrosion resistance on both SS materials. Also, it can be seen clearly that the arc diameter of AISI 316 is larger than that of AISI 304. This indicates that AISI 316 has better corrosion resistance.

Fig. 2c and f show that the slope of the |*Z*|-frequency decreases as the chloride concentration increases. This suggests deviation from the ideal capacitor. This may be attributed to the irregular distribution of the applied potential, which results in the variations of time constants (related to the charge–discharge time of a capacitance).^33^ This may be caused by electrode surface irregularities, surface roughness, fractal surfaces, *etc.*

**Fig. 3:**
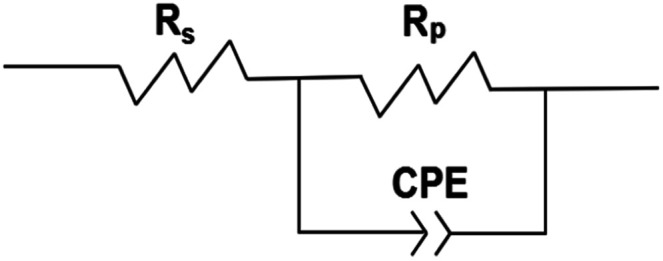
Electrical equivalent circuit for AISI 304 and AISI 316 in simulated solution.

EIS analysis indicates that the electrode surface experiences periodic charging and discharging of the electric double layer. The former is called the non-Faraday process and the latter is called the Faraday process. The impedance of the electrode is equivalent to that of a circuit composed of the non-Faraday impedance (*Z*_NF_) and the Faraday impedance (*Z*_F_) in parallel. Of course, there is also a resistance between the reference electrode and the solution. This is shown in front of the parallel circuit and referred to as the solution resistance. Based on the analysis above, the impedance was fitted using the equivalent electrical circuit depicted in Fig. 3. Traditionally, a capacitor is used to measure the *Z*_NF_. When disturbed by a polarization potential or polarization current, the non-faradaic process from the double layer on the electrode surface is the same as the disturbance signal produced during charging and discharging. Thus, a constant-phase element (CPE) unit was used to account for the frequency dispersion observed *via* the disturbance signals. If diffusion and adsorption are ignored, *Z*_F_ can be replaced by a polarization resistance (*R*_P_). The passivating film *R*_P_ experienced by the current flow is related to the time constant (*τ*) presented at medium and low frequencies. Otherwise, the solution or electrolyte resistance between the reference and working electrodes is represented by *R*_S_.

Typically, a CPE is used to represent the non-ideal capacitance of the electrode when seeking to simulate a real capacitor. Its impedance is defined by the following equation:1
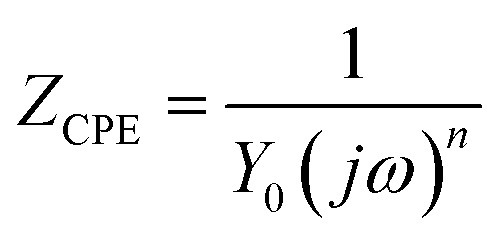
where *Y*_0_ is the admittance, *j*^2^ = (−1), *ω* is the angular frequency, and *n* is a dimensionless fractional exponent. A CPE implies a capacitor, which could be defined by the values of *n*. When *n* = 0 or 0.5, the CPE represents a pure resistor or a Warburg impedance, respectively. If *n* = 1, the CPE is an ideal capacitor without a resistor. The overall impedance according to this model can be determined using the equation below:2
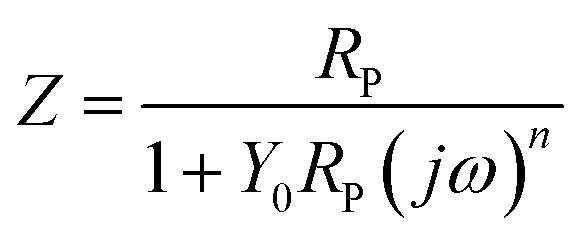
where the parameters have been described previously. EIS results for AISI 304 and AISI 316 in simulated solutions with various chloride concentrations are described in Table 3.

**Table 3:** EIS data for AISI 304 and AISI 316 in simulated solutions

[Cl^−^] (mg L^−1^)	*R*_S_ (Ω cm^2^)	Error (%)	*R*_P_ (MΩ cm^2^)	Error (%)	*Y*_0_ (μS cm^−2^ s^*n*^)	Error (%)	*n*	Error (%)	*χ* ^2^
**AISI 304**									
25	490	1.20	0.608	0.74	69.51	0.68	0.78	0.79	4.9 × 10^−3^
50	359	0.40	0.596	0.31	76.64	0.48	0.80	0.23	2.4 × 10^−3^
100	325	0.84	0.413	0.66	87.46	0.52	0.81	0.62	2.4 × 10^−3^
200	114	0.86	0.245	1.24	133.16	1.73	0.80	0.63	2.1 × 10^−3^
400	89	1.05	0.131	1.15	149.25	0.64	0.79	0.59	2.8 × 10^−3^

**AISI 316**									
25	442	1.20	0.765	0.39	54.62	1.96	0.78	1.08	8.1 × 10^−3^
50	336	1.06	0.672	1.09	77.47	1.70	0.79	0.95	7.0 × 10^−3^
100	217	0.74	0.396	0.47	86.05	1.36	0.82	0.63	3.1 × 10^−3^
200	137	0.84	0.327	0.34	88.51	1.25	0.79	0.52	1.9 × 10^−3^
400	115	1.02	0.172	1.29	107.81	1.69	0.78	0.66	2.6 × 10^−3^

We combine the EIS results in Table 3 with Fig. 2b and e to show that the phase angle peak shifts to lower frequency values. In addition, the capacitive region shrinks substantially when *R*_S_ increases. One can conclude that the phase angle (*ϕ*) is calculated using arctan (*Z*_im_/*Z*_re_), where *Z*_im_ and *Z*_re_ are the imaginary and real parts of the impedance, respectively, and are represented using complex numbers. It can be seen from formula (2) that *R*_P_ is contained exclusively in the impedance function. Thus, *R*_P_ played an important role in the total impedance, and depended on the chloride concentrations.^34^

According to eqn (1), there is an obvious relationship between the CPE and the index *n*. When *n* equals zero, the CPE is equivalent to a pure capacitor. And the values of the capacitor could be determined by *Y*_0_. Then when *n* deviates from zero, the capacitance decreases accordingly. Hsu and Mansfeld^35^ proposed the concept of an effective capacitance (*C*_eff_), which is calculated using eqn (3). It shows how the capacitance changes based on the chloride concentration.

3 *C*_eff_ = *Y*_0_(*ω*_C_)^*α*−1^

where *ω*_C_ is the critical angular frequency (rad s^−1^), which is related to the maximum of the imaginary component of the impedance. It can be calculated based on *Y*_0_, *R*_P_, and *α* from the EIS results using the following equation:4
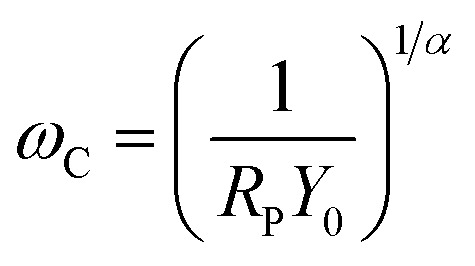


The *α* in eqn (4) can be obtained using the following relations:^36^

5 *E*_corr_ = *A* − *B* log *α*_Cl^−^_

6 *B* = *A* − 2.303*RT*/*αF*

where *A* and *B* are constants that can be determined from Fig. S2.†*R*, *T*, and *F* are the gas constant (8.314 J mol^−1^ K^−1^), absolute temperature (313 K), and Faraday constant (96 485 C mol^−1^), respectively. According to eqn (5) and (6), the critical frequencies for AISI 304 and AISI 316 are 0.0084 and 0.15 Hz, respectively. These the critical frequencies of the samples from the fitting results and are shown in Fig. 2. The *ω*_C_ values for both AISI 304 and AISI 316 are higher than that of experimentally measured, this inferring that there is no change of the passivating film at low frequencies.

Next, *C*_eff_ was calculated according to eqn (3). Comparison of the resulting values to *Y*_0_ in Fig. 5 indicates that there is a marked difference between the two parameters.

**Fig. 4:**
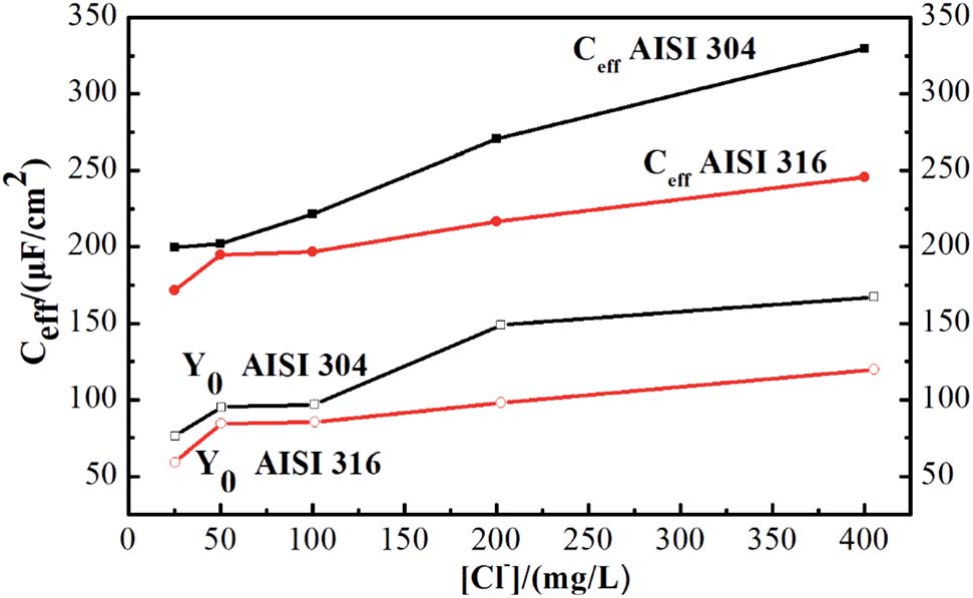
Admittance (*Y*_0_) and calculated effective capacitance (*C*_eff_) of AISI 304 and AISI 316 at different chloride concentration.

The *C*_eff_ of AISI 304 and AISI 316 from Fig. 4 are plotted separately and shown in Fig. S1.† The *C*_eff_ values of both SS samples exhibit similar trends when the chloride concentration changes. Their behaviours can be described using linear fits. In addition, Fig. S1† indicates that the *C*_eff_ of AISI 304 ranges from 200 to 330 μm cm^−2^, while that of AISI 316 ranges from 170 to 230 μm cm^−2^. Obviously, the *C*_eff_ of AISI 316 is consistently lower than that of AISI 304.

**Fig. 5:**
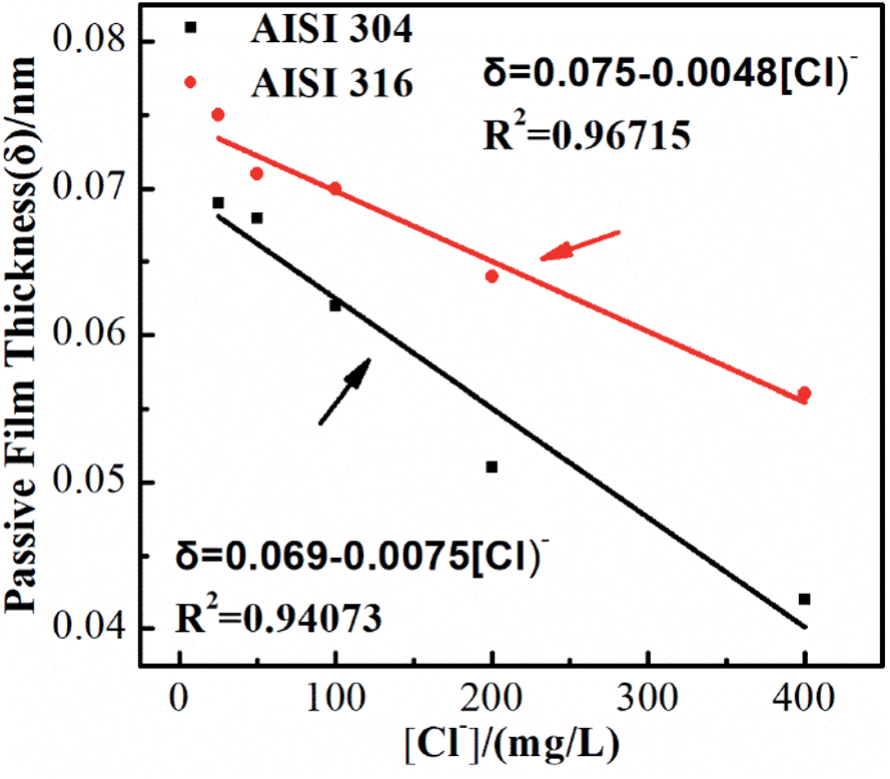
Calculated passive film thickness (*δ*) of AISI 304 and AISI 316 at different chloride concentration.

Treating the capacitive system as a parallel plate capacitor allows the passivating film thickness (*δ*) to be estimated from *C*_eff_ using the following equation:

7 *δ* = *ε*_0_*ε*/*C*_eff_

where *ε*_0_ is the vacuum permittivity (8.854 × 10^−12^ F m^−1^) and *ε* is the dielectric constant (15.6) of the passivating film generated on SS. The metal composition and passivating film thickness all have substantial effects on the corrosion resistance of a metal or a passivating alloy.^37^ If the chemical components of the materials are similar, the thickness of passivating film is the key factor that governs the corrosion resistance. Therefore, for AISI 304 and AISI 316, it is necessary to discuss and study the passivating film thickness. The thicknesses of the passivating films on these two SS materials are calculated using eqn (7) and shown in Fig. 5. There is an obvious negative linear relationship between the thickness and the chloride concentration. This indicates that increasing the amount of chloride in the simulated solution leads to the generation of thinner passivating films, even though the thin film can also protect the SS surface. The slopes of the various lines are related to the corrosion rate for the kinetics consideration. The corrosion rate of AISI 316 is clearly lower than that of AISI 304. This indicates that AISI 316 offers better corrosion resistance. The phenomenon can be attributed to the lower *I*_corr_ exhibited by AISI 316.^38^ Moreover, the thicker passivating film can inhibit ion migration.^39^ This decreases the corrosion rate of AISI 316.

**Fig. 6:**
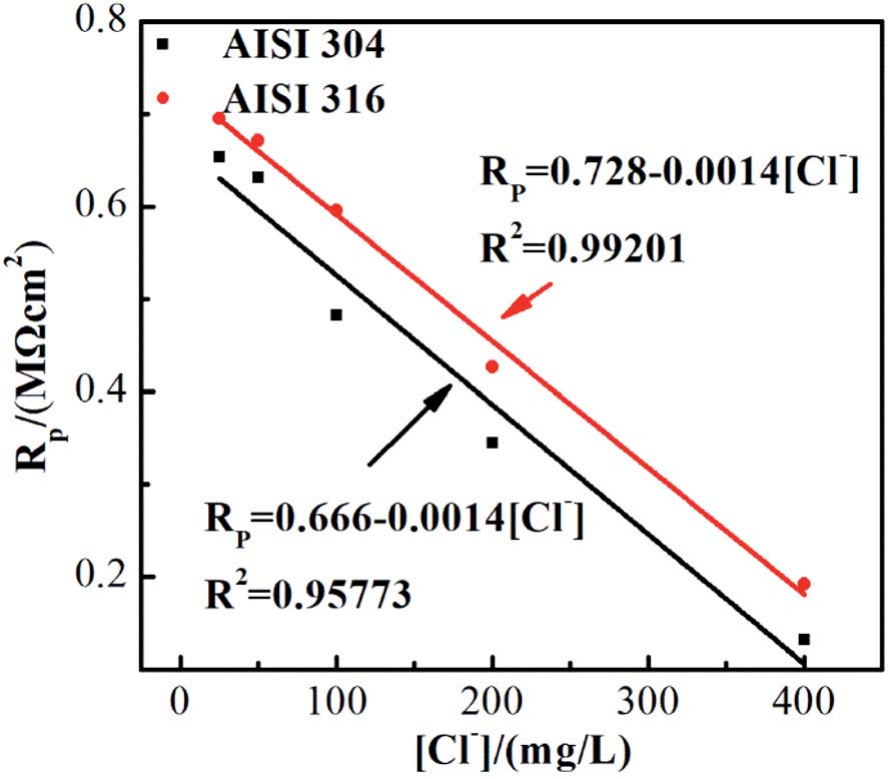
*R*_P_ of EIS experimental data for AISI 304 and AISI 316 at different chloride concentration.

As previously described, *R*_P_ represents the electrical resistance of the passivating film. Fig. 6 shows the *R*_P_ values of the two SS materials in simulated solutions with various chloride concentrations. *R*_P_ decreases as the chloride concentration increases. Linear fitting indicates that the SS *R*_P_ values and chloride concentration have a clear negative linear relationship. It is also noted that the corrosion resistances of these two SS materials decrease at the same rate with respect to the Cl ion concentration, although the resistance of AISI 316 is always higher than that of AISI 304. This phenomenon is consistent with previous studies regarding passivating film thicknesses. The results above demonstrate that the presence of chloride not only affects the corrosion resistance of SS but also affects the protective performance of the passivating film in its non-polarised state.

### 3.2 Potentiodynamic polarization test

**Fig. 7:**
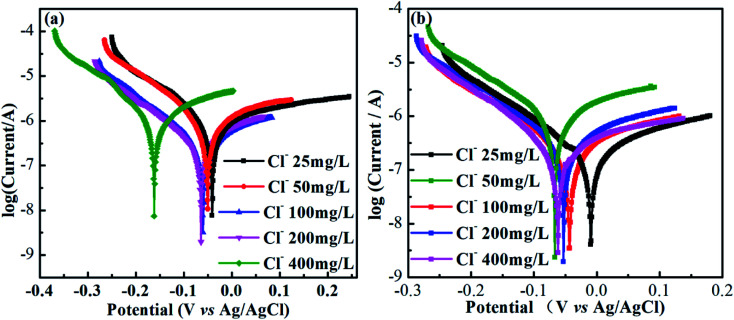
Polarization curves for AISI 304 (a) and AISI 316 (b) at different chloride concentration.

Fig. 7(a) and (b) show potentiodynamic polarization tests (polarization curves) of AISI 304 and AISI 316 in simulated solutions with various chloride concentrations. Obviously, the shapes of the polarization curves in Fig. 7(a) and (b) are quite similar. All of the anodic polarization curves exhibit obvious passivation zones. The largest passivation zone appears at a chloride concentration at 25 mg L^−1^. In general, the length of the passivation zone can reflect the corrosion resistance of the electrode. The larger the interval, the stronger the corrosion resistance. Thus, the corrosion resistances of the two SS samples are highest at a chloride concentration of 25 mg L^−1^.

It also can be seen from Fig. 7(a) and (b) that all of the self-corrosion potentials of AISI 304 and AISI 316 move in the negative direction as the chloride concentration increases. Generally, the more negative the self-corrosion potential, the worse the corrosion resistance. There is an obvious negative movement of the self-corrosion potential at a chloride concentration of 400 mg L^−1^ in Fig. 7(a). This indicates that chloride enrichment can cause serious damage to the AISI 304 surface. For AISI 316, the change in the self-corrosion potential with the chloride concentration is smaller. That implies a stronger corrosion resistance due to the reduced chloride accumulation on the passive film. So, the passivating film was still effective to protect the metal materials.^40^

**Fig. 8:**
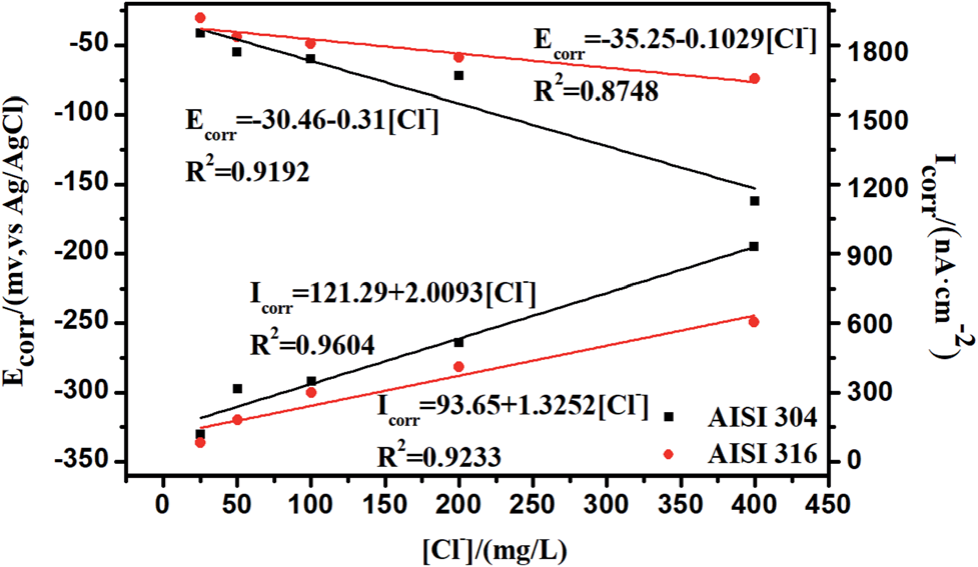
Potentiodynamic polarization test for AISI 304 and AISI 316.

The relationships between *E*_corr_, *I*_corr_, and the chloride concentration are reflected in Fig. 8, where the *E*_corr_ values for the AISI 304 and AISI 316 electrodes are calculated using the cathodic and anodic Tafel slopes from the polarization curve. The corrosion current density (*I*_corr_) is obtained based on the corrosion currents from the potentiodynamic polarization test. The relevant data from Fig. 8 are shown in Table S1.† It can be observed that the *E*_corr_ values of the two SS materials decrease with the chloride concentration, while *I*_corr_ moves to the opposite side with the increase of Cl^−^. In other words, the *E*_corr_ and *I*_corr_ values of the two SS materials have negative and positive linear relationships with the chloride concentration, respectively.

Previous studies indicate^41^ that *R*_P_ is often inversely proportional to the current density a given *E*_corr_. The corrosion current density (*I*_corr_) is related directly to the corrosion rate. Thus, the determination of *I*_corr_ can provide valuable information about the corrosion resistance in a specific environment. In Fig. 6 and 8, there is a clear negative linear relationship between *R*_P_ and the chloride concentration but a positive relationship between *I*_corr_ and the chloride concentration. This is completely consistent with previous studies.^42^*I*_corr_ also has substantial influence on the SS passivating film. The increased corrosion current density in the presence of chloride thins the passivating film, leading to further corrosion.

Comparison of the linear fit data from AISI 304 and AISI 316 in Fig. 8 allows one to determine whether *E*_corr_ or *I*_corr_ depend on the chloride concentration. One can also determine that the *R*^2^ (linear correlation coefficients) of the AISI 304 are always higher than that of AISI 316. Based on this, it can be concluded that the polarization behaviour of AISI 304 is more closely linked to Cl^−^ concentrations, and the passive film is likely to change in chloride. The results indicate that the stability of the AISI 304 passivating film is worse than that of the AISI 316 film. This indicates that AISI 304 is more susceptible to influence from chloride.

### 3.3 SEM analysis

**Fig. 9:**
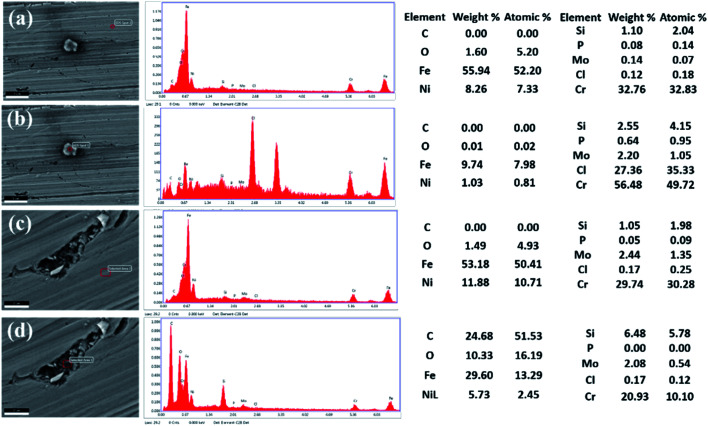
EDS analysis for AISI 304 (a and b) and AISI 316 (c and d) in different positions.

Scanning electron microscopy (SEM) was used to observe the microstructures of the two SS electrodes before and after the potentiodynamic polarization tests. Comparison of the microstructures of AISI 316 and AISI 304 (Fig. S3(a) and (b)†) indicates that there are various corrosion pits on the AISI 304 and AISI 316 surfaces after processing in 200 mg L^−1^ Cl^−^ solutions. To analyse the corrosion mechanism further, EDS (Energy Disperse Spectroscopy) was used to perform elemental analysis of the corrosion products. From Fig. 9(a) and (b), it showed that the chlorine and chromium concentrations are higher around the corrosion pits than that on other areas for AISI 304. But the iron and oxygen contents have the opposite phenomena. The results indicated that accumulation of Cl^−^ encouraged the occurring of corrosion, where Fe and Cr are the main elements that react with Cl^−^. Some corrosion products derived from Fe and Cl^−^ might be lost, but the products from the reaction between Cr and Cl^−^ typically accumulate around the corrosion pit.

In the EDS analysis of AISI 316 shown in Fig. 9(c) and (d), the variation of Fe and O is similar to that of AISI 304, where the contents decreased when the sample was corroded. However, the chlorine contents are close (about 0.17 wt%) regardless of whether the AISI 316 is corroded or not. This is different with AISI 304, where the Cl content had a marked increase from 0.12 wt% to 27.36 wt% when the sample was corroded. The EDS analysis indicates that corrosion can occur as a reaction between Cl^−^ and several components such as Fe, Cr, and O ions. Some reaction products tend to exist in free state, while others apt to aggregate around the corrosion areas. In addition, AISI 304 is more likely to be corroded due to Cl^−^ enrichment on its surface.

## 4 Mechanism analysis

**Fig. 10:**
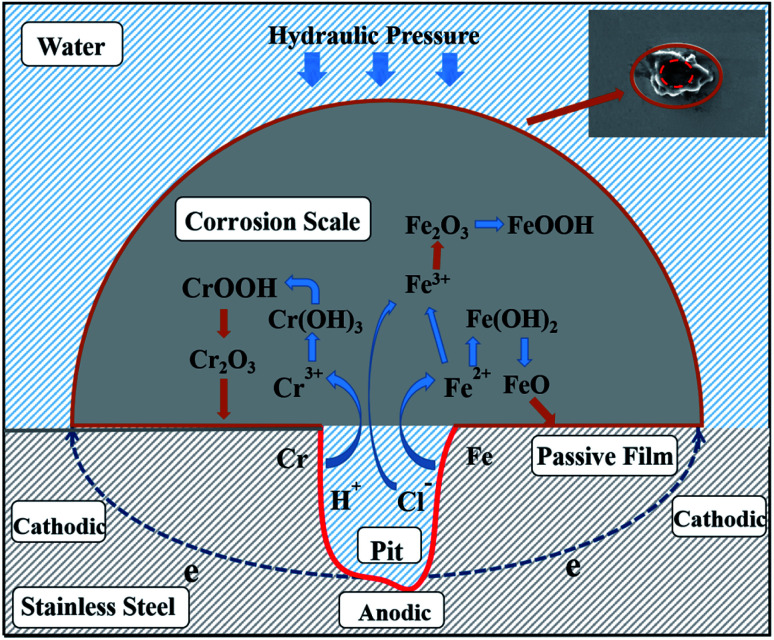
Hypothesized model sketch of formation mechanism of SS corrosion scale.

SEM was used to observe the sample microstructures. Corrosion is typically caused by chemical and electrochemical reactions^43^ and is usually uniform.^44^ It has been proven that chloride is the major factor in the SS corrosion process. The SS corrosion model is deduced and shown in Fig. 10 based on the analysis above and related phenomena. Chloride attaches to the passivating film by dissolving its surface. Next, some etched pits appear on the passivating film. These can be attributed to the anodic reactions shown below:

8 Fe = Fe^2+^ + 2e^−^

9 Cr = Cr^3+^ + 3e^−^

In a chloride-rich solution, the increased Fe^2+^ and Cr^3+^ in the etch pits can cause continuous migration of chloride to maintain a relatively neutral solution.^45^ The solution becomes acidic and its oxidation state changes such that some Fe^2+^ can be oxidised to Fe^3+^. Then, metal chloride is generated due to the combination of Fe^2+^ and Fe^3+^ with chloride ion.^46^ Next, these metal chlorides are hydrolysed into metal hydroxides and hydrogen ions by water. This series of reactions accelerates corrosion development and can be explained using the following chemical formulas:

10 Cl^−^ + H_2_O = HClO + H^+^ + 2e^−^

11 Fe^2+^ + 2Cl^−^ = FeCl_2_

12 Cr^3+^ + 3Cl^−^ = CrCl_3_

13 2FeCl_2_ + 2HClO = 2FeCl_3_ + H_2_O

14 FeCl_2_ + 2H_2_O = Fe(OH)_2_ + 2H^+^ + 2Cl

15 FeCl_3_ + 3H_2_O = Fe(OH)_3_ + 3H^+^ + 3Cl^−^

16 CrCl_3_ + 3H_2_O = Cr(OH)_3_ + 3H^+^ + 3Cl^−^

Some of the Fe(OH)_2_ produced *via*eqn (14) can dehydrate and become FeO as follows:

17 Fe(OH)_2_ = FeO↓ + H_2_O

The Fe(OH)_3_ produced *via*eqn (15) and Cr(OH)_3_ produced *via*eqn (16) can be converted into FeOOH and CrOOH *via* dehydration. Then, FeOOH and CrOOH continue to dehydrate and generate Fe_2_O_3_ and Cr_2_O_3_. Fe_2_O_3_, Cr_2_O_3_, FeOOH, and CrOOH precipitate as corrosion scale and become the main components of the corrosion products. The related chemical reactions can be depicted as follows:

18 Fe(OH)_3_ = FeOOH↓ + H_2_O

19 2FeOOH = Fe_2_O_3_↓ + H_2_O

20 Cr(OH)_3_ = CrOOH↓ + H_2_O

21 2CrOOH = Cr_2_O_3_↓ + H_2_O

In summary, the occurring of corrosion is mainly processed as the formation of corrosion pits on the passive film. The reaction results in a continuous accumulation of corrosion products due to the invading of chlorine. Electrochemical reactions between the iron and chromium phases generated play crucial roles in the corrosion process, where the absence of oxygen suppresses AISI 316 corrosion. The results indicate that AISI 316 pipelines can provide a reliable means of delivering reclaimed water. The electrochemical analysis indicates that the bottom of the pit can be seen as the anode and the passivating film on the SS surface can be seen as the cathode. Electrons are transferred directly from the anode to the cathode on the SS substrate when a chloride-containing solution is used as the electrolyte. Finally, corrosion products with a wavy appearance formed under the action of water pressure.

## 5 Conclusions

In this study, electrochemical tests were performed using various chloride concentrations in order to evaluate the corrosion resistances of AISI 304 and AISI 316 pipeline materials. The focus was on the influence of chloride. The EIS results indicated that the two SS materials are highly similar. They exhibited resistive behaviour in Bode plots. The Nyquist plots indicated that AISI 316 had stronger corrosion resistance than AISI 304. Also, there was a positive correlation between *C*_eff_ and the chloride concentration. The passivating film became thinner as the chloride concentration increased. Moreover, the chloride concentrations were correlated linearly with the *R*_P_ values. This was confirmed using the polarization curve results. *E*_corr_ decreased and *I*_corr_ increased as the chloride concentration increased. Finally, the SEM results demonstrated the critical role of chloride in corrosion of the passivating film and further confirmed that AISI 316 has better corrosion resistance than AISI 304. This provides a basis for further study of corrosion resistance among SS heating pipe networks during industrial reclaimed water transportation.

## Conflicts of interest

There are no conflicts to declare.

## Supplementary Material

RA-011-D1RA06695A-s001

## References

[cit1] Barker Z. A., Stillwell A. S. (2016). Environ. Sci. Technol..

[cit2] Meneses M., Pasqualino J. C., Castells F. (2010). Chemosphere.

[cit3] Wester J., Timpano K. R., Cek D., Broad K. (2016). Water Resour. Res..

[cit4] Zhang H. Y., Tian Y. M., Kang M. X., Chen C., Song Y. R., Li H. (2018). Chemosphere.

[cit5] Annus I., Vassiljev A., Kandler N., Kaur K. (2020). J. Water Supply: Res. Technol.--AQUA.

[cit6] Diaz B., Swiatowska J., Maurice V., Seyeux A., Normand B., Harkonen E., Ritala M., Marcus P. (2011). Electrochim. Acta.

[cit7] Huy D. H., Seelen E., Liem-Nguyen V. (2020). J. Water Process. Eng..

[cit8] Xu X. Y., Liu S. M., Liu Y., Smith K., Cui Y. (2019). Eng. Failure Anal..

[cit9] Martin U., Bosch J., Ress J., Bastidas D. M. (2021). Constr. Build. Mater..

[cit10] Munis A., Zheng M., Zhao T. (2020). Mater. Chem. Phys..

[cit11] Yu Z., Lu J., Chen M., Wang J., Wang F. (2021). Corros. Sci..

[cit12] Li X., Chang L., Liu C., Leng B., Ye X., Han F., Yang X. (2021). Corros. Sci..

[cit13] Gong K., Wu M., Xie F., Liu G. X., Sun D. X. (2021). Mater. Chem. Phys..

[cit14] Zheng C., Zhang C., Wang X. Y., Gu J. (2021). Anti-Corros. Methods Mater..

[cit15] Ofoegbu S. U. (2021). Materials.

[cit16] Hou B., Li X., Ma X., Du C., Zhang D., Zheng M., Xu W., Lu D., Ma F. (2017). npj Mater. Degrad..

[cit17] Lyu S. D., Chen W. P., Zhang W. L., Fan Y. P., Jiao W. T. (2016). J. Environ. Sci..

[cit18] Hu J., Dong H. Y., Xu Q., Ling W. C., Qu J. H., Qiang Z. M. (2018). Water Res..

[cit19] Lin X., Xu Q., Li Y., Zhao B., Li L., Qiang Z. (2021). J. Environ. Sci..

[cit20] Bruton T. A., Sedlak D. L. (2018). Chemosphere.

[cit21] Samiee R., Ramezanzadeh B., Mahdavian M., Alibakhshi E. (2019). J. Cleaner Prod..

[cit22] Wang C., Chen J. X., Hu B. S., Liu Z. Y., Wang C. B., Han J., Su M., Li Y. H., Li C. L. (2019). J. Cleaner Prod..

[cit23] Peng C. Y., Korshin G. V., Valentine R. L., Hill A. S., Friedman M. J., Reiber S. H. (2010). Water Res..

[cit24] Yang F., Shi B. Y., Gu J. N., Wang D. S., Yang M. (2012). Water Res..

[cit25] Ding J. W., Tang B., Li M. Y., Feng X. F., Fu F. L., Bin L. Y., Huang S. S., Su W., Li D. N., Zheng L. C. (2017). J. Cleaner Prod..

[cit26] Keramat A., Zanganeh R. (2019). J. Water Supply: Res. Technol.--AQUA.

[cit27] Liu Y. Q., Song Z. J., Wang W. Y., Jiang L. H., Zhang Y. J., Guo M. Z., Song F. Y., Xu N. (2019). J. Cleaner Prod..

[cit28] Sander A., Berghult B., Broo A. E., Johansson E. L., Hedberg T. (1996). Corros. Sci..

[cit29] Dornhege M., Punckt C., Hudson J. L., Rotermund H. H. (2007). J. Electrochem. Soc..

[cit30] Tian W. M., Du N., Li S. M., Chen S. B., Wu Q. Y. (2014). Corros. Sci..

[cit31] Allison A., Andreas H. A. (2019). J. Power Sources.

[cit32] Ekm A., Azm B., Nca B., Mogpb C., Gtc D., Mj E. (2020). Constr. Build. Mater..

[cit33] Zhang X. H., Zhang X., Sun X. Z., An Y. B., Song S., Li C., Wang K., Su F. Y., Chen C. M., Liu F. Y., Wu Z. S., Ma Y. W. (2021). J. Power Sources.

[cit34] Lou X. Y., Singh P. M. (2011). Electrochim. Acta.

[cit35] Hsu C. H., Mansfeld F. (2001). Corrosion.

[cit36] Lin L. F., Chao C. Y., Macdonald D. D. (1981). J. Electrochem. Soc..

[cit37] Grgur B. N., Lazic V., Stojic D., Rudolf R. (2021). Corros. Sci..

[cit38] Bosch J., Martin U., Ress J., Klimek K., Bastidas D. M. (2021). Appl. Sci..

[cit39] Kong D., Dong C., Xu A., He C., Li X. (2017). Corros. Eng., Sci. Technol..

[cit40] Abreu M. C., Cristóbal J. M., Losada R., Nóvoa R. X. (2004). J. Electroanal. Chem..

[cit41] Fajardo S., Bastidas D. M., Criado M., Bastidas J. M. (2014). Electrochim. Acta.

[cit42] Dastgerdi A. A., Brenna A., Ormellese M., Pedeferri M. P., Bolzoni F. (2019). Corros. Sci..

[cit43] Sabará C. V. L., Prachedes L. N. S., Santos L. C., Sabará M. A., Souza R. C., Sene A. F., Caldeira L., Vaz G. L., Oliveira J. R., Gomes J. A. C. P., Bueno A. H. S. (2021). Eng. Failure Anal..

[cit44] Nie J., Wei L., Jiang Y., Li Q., Luo H. (2021). Mater. Today Commun..

[cit45] Amo A., Aao B., Aio A., Nt A., Sur A. (2020). Corros. Sci..

[cit46] Palogi C., Parthasarathy S. M., Srinivasan R. (2021). Prog. Nucl. Energy..

