# Supplementary material for: Chloride corrosion behavior on heating pipeline made by AISI 304 and 316 in reclaimed water
Source: RSC Adv. 2021 Dec 2;11(61):38765–73. doi: 10.1039/d1ra06695a (PMC9044296; doi:10.1039/d1ra06695a)
Supplement: RA-011-D1RA06695A-s001 [file RA-011-D1RA06695A-s001.pdf]

Electronic Supplementary Material (ESI) for RSC Advances.
This journal is © The Royal Society of Chemistry 2021

Supporting Information

Chloride corrosion behavior on heating pipeline made by AISI 304 and 316 in reclaimed water

Xi Chen,[a] Hongyan Liu, [a] Xiang Sun,[a] Botao Zan [b] and Meisheng Liang *[a]

[a]College of Environmental Science and Engineering, Taiyuan University of Technology, Taiyuan, China.

[b]University of Washington, Seattle, 98105, WA, USA

07/12/2021, the ESI was first published 02/12/2021.

This version of the Electronic Supplementary Information replaces a previous copy in which the author order was incorrect.

S1. The detailed for electrochemical test

In order to reach a stable state for the experimental system before potentiodynamic polarization tests and EIS measurements, the open circuit potential method was used to monitor the corrosion potential (E_{corr}) of each sample for 30 min. Then EIS measurements were initially performed because its weak influence to working electrode, and the date were recorded using E_{corr} in a frequency range from 10^5 Hz to 10^{-2} Hz with a sweeping frequency range of 12 points per decade frequency. After recording, EIS spectra were fitted by Z-view software. Finally, the potentiodynamic polarization tests with potential scan rate of 0.0005 V s^{-1} were carried out for analyzing the pitting corrosion susceptibility of working electrode, where the potential value was defined as the pitting potential (E_{pit}).

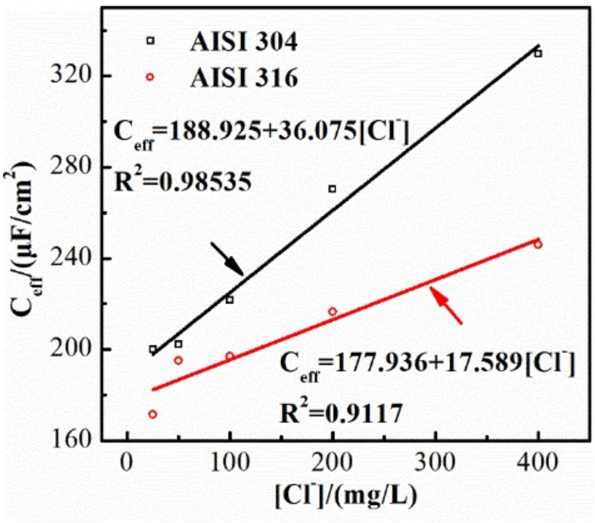

Fig. S1 Calculated effective capacitance (C_{eff}) of AISI 304 and AISI 316 at different chloride concentration.

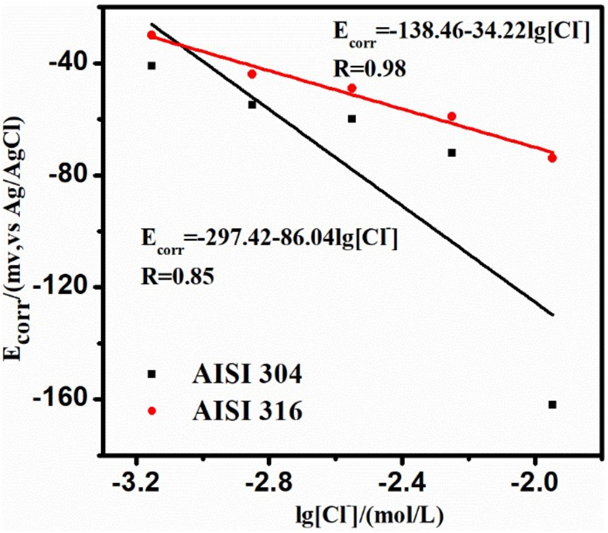

Fig. S2 Corrosion potential (E_{Corr}) as a function of the Cl^- concentrations for AISI 304 and AISI 316.

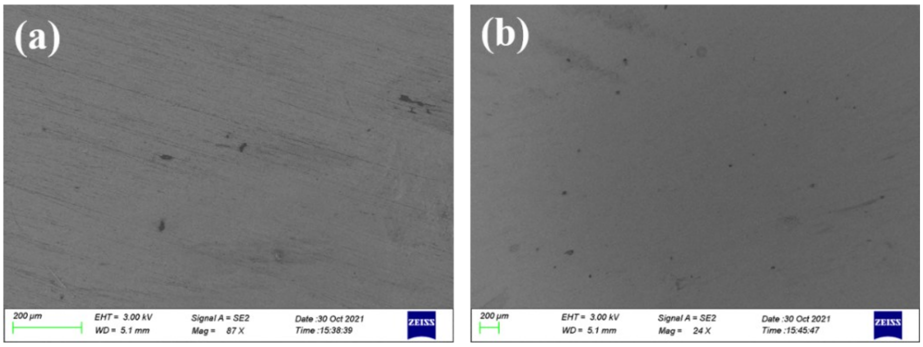

Fig. S3 SEM images of AISI 304 (a) and AISI 316 (b) after being corroded in 200 mg/L of Cl^-.

Table S1 I_{Corr} and E_{Corr} data for AISI 304 and AISI 316 in potentiodynamic polarization test

Cl^-/(mg/L)	25	50	100	200	400
I_{Corr}/304(nA • cm^2)	-41	-55	-60	-72	-162
I_{Corr}/316(nA • cm^2)	-30	-44	-49	-59	-74
E_{Corr}/304(mV)	100	298	334	506	926
E_{Corr}/316(mV)	60	162	284	395	595
